# An intervention development study of an mHealth app to manage women’s health and safety while on probation

**DOI:** 10.1186/s40352-024-00277-6

**Published:** 2024-05-21

**Authors:** Allison D. Crawford, Emily J. Salisbury, Jacqueline M. McGrath

**Affiliations:** 1 School of Nursing, The University of Texas Health at San Antonio, 7703 Floyd Curl Drive, San Antonio, TX 78229 USA; 2 College of Social Work, The University of Utah, 395 1500 E., Salt Lake City, UT 84112 USA

**Keywords:** Community supervision, Probation, Sexual health, Interpersonal violence, MHealth, Self-efficacy, Safety, Women, Technology

## Abstract

**Purpose:**

Preliminary studies have suggested that women are responsive to using technology to manage their health, due to its discreet, convenient, and cost-effective nature. Yet, there are limited mobile health (mHealth) apps specific to women’s needs, particularly those on probation. The purpose of this study was to explore features of 2 existing mHealth applications related to sexual health and safety, specific to interpersonal and sexual violence, to answer research questions related to the usability, barriers, and facilitators of mHealth app use for women on probation.

**Subjects:**

We purposefully sampled from a local adult probation site and utilized snow-ball sampling to recruit 11 women who were on probation and owned iPhones.

**Methods:**

We conducted an exploratory intervention development study using a qualitative design. Social Cognitive Theory was used for data synthesize and organization.

**Findings:**

Three themes emerged: (1) *It made me take time for myself*; (2) *It helped me to be more respectful of my body*; (3) *The connectivity….that was helpful.*

**Major implications:**

Participants expressed mHealth apps to be usable, feasible, accessible and promoted self-efficacy by allowing them track symptoms and patterns of behavior specific to health and safety in a discreet, convenient, and effective manner. This research suggests that a culturally tailored mHealth app may be an appropriate intervention to provide timely gender-responsive feedback, resources, and health care to women on probation.

## Background

The United States (U.S.) has witnessed a troubling rise in the number of women that experience incarceration due to increasingly punitive criminal sentencing practices and policy decision-making (Heimer et al., 2023). Over the past 40 years, the female population in U.S. prison systems has surged by 834%, rendering women the fastest-growing segment of the incarcerated population (Wagner & Sawyer, 2018). This surge underscores a concerning trend, with factors such as harsh drug sentencing policies, limited access to mental health services and treatment for substance use disorder, as well as systemic social and economic inequalities playing pivotal roles.

Parallel with the notable rise in the incarceration rate of women, there has been a twofold increase in the number of women managed through community supervision since the early 1990s (Pew Charitable Trusts, 2018). Women comprise approximately one-quarter of the 3.9 million adults under supervision in the U.S. (Kaeble, 2021), with probation encompassing the largest portion of women engaged with the legal system (Morash & Hoskins, 2022). As such, three out of four women involved with the legal system are released from jail (or prison) on probation to serve-out the criminal sentence (Kajstura & Sawyer, 2023). A recent report from the Bureau of Justice Statistics (2020) revealed that over 700,000 women were on probation, with an additional 100,000 women on parole after serving prison sentences (Kaeble, 2021).

Most women involved with the legal system are rerouted from formal incarceration and placed on probation. These women are more likely to need access to health care services and resources within the community versus a jail or prison system (Lorvick et al., 2022). Although probation is considered less disruptive than incarceration, many women have described social and economic barriers that inhibit access to appropriate healthcare services, which may lead to higher rates of morbidity, mortality, and rearrest (Hawks et al., 2020; Lorvick et al., 2022). Barriers or contextual issues that may interfere with their health are often related to unsafe housing, limited transportation and childcare, the inability to obtain health insurance, and fear of stigma or rearrest (Crawford et al., 2022a, 2023c).

Despite probation being the most prominent form of correctional oversight for women of childbearing age, this population remains understudied. Women on probation have similar inequities as women in prison, such as heightened legal oversight and poorer social determinants of health with amplified morbidity and mortality (Hawks et al., 2020; Lorvick et al., 2022). In addition, women on probation have context specific conditions such as interpersonal and sexual violence, sexual coercion, and forced sex work to survive within the community, which may influence their overall health (Crawford et al., 2022a) (Prost et al., 2022). Consequently, there is an increasing need to address the sexual health and safety of women on probation. Over 70% of women are rearrested and return to jail for technical violations within their first three years on probation (De Rooy et al., 2019). Some recurrent arrests appear to be related to limited access to community-based health services that can be somewhat more easily accessed while incarcerated (De Rooy et al., 2019; Sawyer, 2019). Further, these women often must choose between meeting the time and financial obligations of their probation sentence versus managing their health to avoid getting re-arrested for a technical violation (Crawford et al., 2022a; (De Rooy et al., 2019). As a result, women within the first year of probation have been noted to have higher emergency department encounters and hospitalizations with decreased use of routine and preventive care services (Lorvick et al., 2022).

With rising smart phone ownership and advances within mobile application technologies, mobile health (mHealth) interventions have demonstrated promising effects on prevention, diagnosis, treatment, and self-management of health conditions (Armaou et al., 2020; Kim et al., 2022). MHealth applications are effective in electronically transmitting health care services and resources to hard-to-reach populations (Kim et al., 2022; Armaou et al., 2020). Hard-to-reach populations such as those in rural communities or those with stigmatizing conditions such as those related to sexual or reproductive health and safety have demonstrated improvements in their health and ability to access appropriate healthcare with use of mHealth technology (Armaou et al., 2020; Crawford et al., 2023a). Preliminary studies have suggested that women are responsive to using technology to manage their health, due to its discreet, convenient, and cost-effective nature. Yet, there has been limited mHealth apps specific to the needs of women on probation (Crawford et al., 2023a).

## Methods

Therefore, we conducted an exploratory study as a means of facilitating future intervention development. This work is the next logical step in our research trajectory (Crawford et al., 2022a, b) that focused on the existing gaps by first using a qualitative design to explore the perspectives of women on probation related to the features of two existing mHealth applications, Sex Tracker by Nice and uSafeus (Julian, 2023; Potter et al., 2020, 2022), that were initially designed for women in college. Our purpose was to answer the following research question: What is the usability, the barriers and facilitators to using the mHealth apps to manage your health and safety?

### Sample

Following approval from the Institutional Review Board (IRB) (23–0076 H), recruitment began in May 2023 and concluded in October 2023. Inclusion criteria were women, ages 18 to 50 years, English speaking, reside in Texas, own iPhones, and on adult probation. We chose to limit inclusion to only iPhone users due to the Nice Sex Tracker app having limited ability to be downloaded on Android phones. The principal investigator (PI) met with stakeholders at the adult probation department prior to recruitment to garner their support and review inclusion criteria. Fliers were distributed to the department and their officers to share with their clients. Women were urged to contact the PI by telephone to be screened for eligibility; a process that took approximately 10 min. Snowball sampling strategies which consisted of word-of-mouth referrals amongst participants were also used to augment the sampling process.

A total of eleven women were recruited to participate in the study. Of these, two participants were recruited by snow-ball sampling. One participant did not complete the study leaving ten (*N* = 10) participants that completed all stages of the study’s protocol. Once eligibility was confirmed, each participant was read the study’s protocol and consent. Consent was provided verbally over the telephone, with information sheets given to each participant explaining the risks, benefits, and methods to request more information, follow-up, or withdrawal from the study electronically.

### Data collection

Data collection consisted of three stages: (1) pre-intervention demographic data; (2) 30-days of interacting with the two mHealth apps; (3) post-intervention qualitative data. The pre-intervention demographic data was collected over the telephone and deidentified. We asked participants question such as their age range, marital status, arrest history, and any history of abuse or violence. We also asked what issues related to their reproductive health were most important to them such as preventing an unplanned pregnancy, sexual violence, sexually transmitted disease (STD), lack of resources or bodily autonomy. We then instructed participants to engage with each app for 30 days. Additional resources within the information sheet were given to the participants such as numbers to call if they experienced any forms of violence, threat, or health-related issues during the 30-days of using the mHealth apps. The PI also had a plan to protect human subjects by mandatory reporting if there were any disclosure during the interviews of violence or threat to the participants or vulnerable persons. We told participants to open each app and all its features at least once to get familiar with the app, however, only use the app’s features as needed. After the 30 days of interacting with the 2 apps, the PI recontacted the participants by text message using Tiger Connect, a university approved text messaging platform to schedule a post-intervention qualitative interview. Once the meeting was scheduled, the PI would call the participants using the telephone to obtain the post-intervention qualitative interview, a process that took approximately an hour. Interviews were completed on the telephone without use of video to maintain participant confidentiality. Probe questions were used to explore usability, barriers, and facilitators of the mHealth applications as identified in the research questions. All interviews were recorded using two different voice recorders and transcribed verbatim by a third-party University-approved transcription service. After transcription, all identifying information was removed.

Particularly because of the vulnerability of this population, intentional steps were taken to protect participant safety and confidentiality throughout data collection. No identifying information was retrieved. Telephone numbers were collected only for retention purposes. Participants were given an unidentified, unique, numerical code for organizational purposes. All study data were kept on a separate password protected university-issues computer and a secure cloud server and stored in a locked room that was only accessible to the PI.

### Apparatus and instruments

Participants were trained by research staff on how to use both apps after the initial app download over the telephone and again in-person when they were given their baseline incentive on day-one of interacting with the mHealth apps. When the participants met with the research team member in person, the research team used the teach-back method to make sure the participants knew how to engage with the apps. Further, the team member would practice with each participant the feature of programming numbers into the uSafeUS app to illicit a fake text or phone call. Throughout the duration of the 30-days both the PI and their research student assistant were available by phone or text to answer any technical questions regarding the mHealth app use. Two participants contacted the research team throughout the 30-day period asking for more clarification. There was also use of text message reminders send through Tiger Connect after one week of mHealth app use to check if participants had any questions, reminding them to engage with the apps, and thanking them for their participation.

One app to track daily sexual behavior, Sex tracker by Nice (Julian, 2023), and one app during times of imminent threat or violence, uSafeUS (Potter et al., 2020, 2022). Both mHealth apps are available free to download and compatible for iPhone users. More detail about these mHealth apps can be found in Table 1. The post-intervention qualitative interview (see Table 2) was guided by the Social Cognitive Theory (SCT) (See Fig. 1) (Bandura, 1986, 1999). The SCT which posits that individuals initiate and maintain behavior by having personal, behavioral, and environmental factors interacting interchangeably (Bandura, 1986, 1999). Ultimately this framework suggests that agency is a motivating factor supporting self-management behavior and assisting individuals to strive for this agency using goal oriented motivational processes (Bandura, 1986, 1999).

**Table 1:** Features of the mHealth apps

#	mHealth App	Frequency of App Use	Features of the App
1	uSafeUS	Acutely, during times of imminent threat or violence	• Designed for college women to assist in obtaining help during times of violence, imminent harm, and resources specific to sexual/dating violence. • The app developer agreed to tailor the app for our participant population with a default tab named “demo college” for our participants for study use. • When participants accessed, they were able to use features such as triggering fake calls or texts to give them a reason to leave an area. Another feature was to alert trusted friends or family when participants did not get home or to their destination on time.
2	Nice Sex Tracker	Daily, as applicable	• Tracks sexual partners, locations, activities, and STDs in a discreet and nonjudgmental style. • Provides analytics of sexual health and behaviors and provides resources information, where to get STDs testing. • Participants were instructed to interact with app daily. There are tabs within the app to input time, activities, partners, location, and barrier methods within sexual encounters. • Tracking also includes sexual intimacy activities, lab or test results, and information about partners.

**Table 2:** Interview guide using social cognitive theory as the guiding framework

#	Question Type	Question & Aims
1	Personal/ Individual Factors	• Describe how you felt when using the [app name] mHealth app (i.e.: comfort, support, frustration, empowered, etc.).? • What assisted you or was a barrier in using the app for the first time; from continuing to you the app? • What would make the app more appealing or usable for you? (colors, fonts, representation) • What do you think has to be addressed to make something like this easier for you to use? Such as training on how to use the app?
2	Behavioral Factors	• Were you motivated to use the app? What motivated you to use the mHealth app such as a desire to be in control of your health, your role as a caregiver, mother, hope for the future etc.? • If you were not motivated to use the app, can you describe the reasons such as: inconvenience, not interested, do not like technology etc.?
3	Environmental Factors	• Describe what it was like using the [name of app] within your environment such as at home, at work, in the community (park, vehicle, on the street, in waiting rooms, appointments, etc.). • What assisted or became a barrier to using the app in these places? • What do you think has to be addressed to make something like this easier for you to use? Such as assistance with cell phone plans, restrictions on data sharing/mandatory reporting; training for agencies who use this? • What concerns do you have with people or systems (PO, probation, counselors, HCPs) having access to the app, your data, and knowing you use this app? • Are there features or data you should have access to while having other pieces of the data be restricted?
4	Personal/ Individual Factors Behavioral Factors	• Tell me how using the [name of app] improved how you felt about managing your health and safety? • Was there anything within the app that decreased your feelings of being capable of managing your health and safety? • Which health and safety features did you find most/least useful? • How did your health/safety improve or decline while using the app?
5	Personal/Individual Factors	• Can you describe what ways your knowledge about your sexual health and safety increased while using [name of app]? • What personal or environmental factors helped with increasing your knowledge such as previous experience using mHealth apps; prior experience managing these health issues; training opportunities; systems allowing you to use the app etc.?
6	Personal/Individual Factors	• How did the use of [name of app] help manage your daily/ emergency/ acute sexual health behaviors? Such as behaviors in safe sex practices; increasing agency and autonomy during sexual encounters; tracking symptoms of STI/STD; tracking; reporting; or identifying instances of imminent or experienced sexual violence? • What outcomes would you like more assistance with and that are most important to you?
7	Personal/Individual Factors	• How did the use of [name of app] assist in accessing resources or support specific to your sexual health and safety? • What concerned you about your confidentiality with the legal, court, CPS, or MAT system, with your partner or family? • How was your health improved or restricted when accessing these resources and support system?

**Fig. 1:**
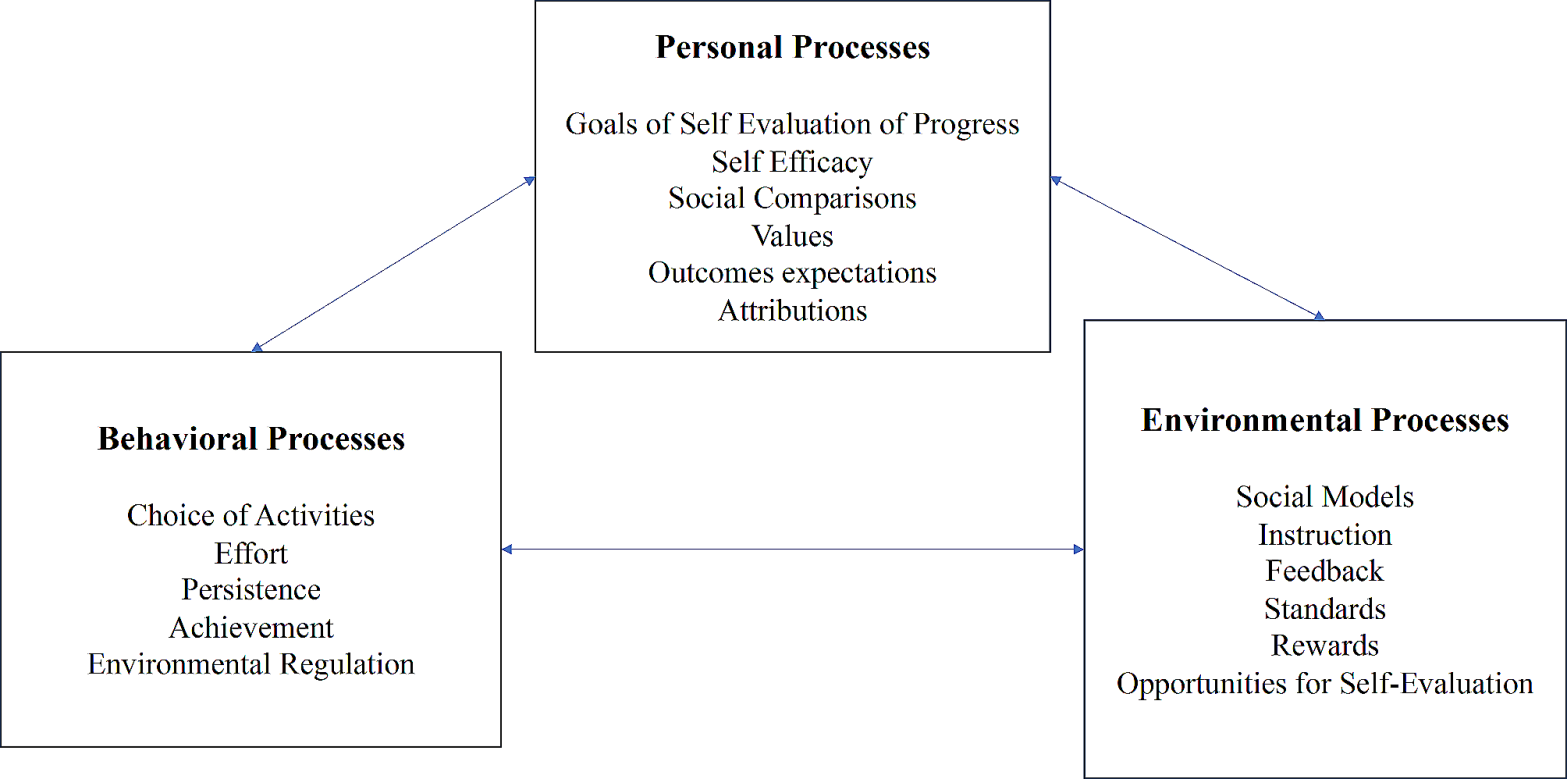
The social cognitive theory Posits that (1) *Personal factors* such as goals can be motivating factors for self-efficacy and behavior change. Goals help individuals focus efforts at a task and by allowing the opportunity to observe and evaluate one’s progress, will lead people to be persistent to their desired goal. (2) *Behavioral factors* that motivate outcomes and self-efficacy are having the ability to have agency in activity, effort, persistence, achievement, and environmental regulation. (3) *Environmental factors* such as programming, accessibility to interventions, peer support, and feedback, can affect an individual’s outcomes and self-efficacy

### Data analysis

Once the qualitative data was transcribed, we began with content analysis by reading the transcripts line by line to become familiar with the data (Elo et al., 2014). The first level of coding consisted of codes for participants’ words and phrases specific to SCT concepts (Bandura, 1986, 1999). These codes were put into the margins of the transcribed interviews using Word. The second level of coding used direct quotes from the participants to develop themes from the coded text matched with SCT concepts. The second level coding data were placed in a table using Word to categorize and track the codes. Codes and themes were reviewed and refined by modifying which theme would serve as the exemplar by two research team members. When there were disagreements, continued discussion occurred until consensus was achieved. There was no use of qualitative analysis software.

## Results

### Pre-intervention baseline data

All the participants disclosed they used the Nice app most compared to the uSafeUS app due to its ability to track daily sexual behavior using diary entries. The participants disclosed they liked the features of the uSafeUS app, however, did not need to utilize them over the 30-day period except for two participants. Nine participants were 26 years of age, heterosexual (*n* = 10) with one identifying as bisexual, not employed (*n* = 8), living in apartments (*n* = 7), had some college (*n* = 7) or were college graduates (*n* = 4). Seven of the participants had at least 2 children with ages ranging 2 months to 24 years old. Nine participants had felonies (*R* = 1–3) with all (*N* = 11) having at least 1 misdemeanor (*R* = 1–13) and had been to county jail (*R* = 1 day to 6 months). Two had been to state prison (*R* = 6 mo-4 years). Three participants had experienced medication for opioid use disorder (*R* = 1–3) in their past. All (*N* = 11) were on community supervision for an average of 2 years (*R* = 1–8 years, *M* = 6 years).

When asked what was most concerning about their health, unintended pregnancy was highest on the list (*n* = 7), next reported was sexual violence and lack of resources (*n* = 6), and then STDs and lack of agency were of a concern (*n* = 4). Six of the participants had engaged in sex work in their past to survive working as escorts (*n* = 3) in the community or staying in romantic/sexual partnerships (*n* = 3) for survival. Seven participants experienced sexual violence or abuse in their past, prior to being involved in this study. Table 3 discusses the frequency and reasons they did not report this violence or abuse. Only three participants had used apps to manage their health such as tracking their heart rate, period, medications, and sobriety. The rest of the sample (*n* = 8) who had never used mHealth app discussed being unaware this technology existed.

**Table 3:** Length of abuse/violence and reasons for not reporting

Length of time of the abuse for each (*n* = 7) participant	Reason for not reporting
20–30 times	Scared, last time was 4 years ago while on probation.
2 times	Scared of more violence. It has affected her mental health till this day.
3 times	She tried, but the perpetrator was her mom’s boyfriend. When she told her family, nobody believed her. She was on parole and her parole officer was the only one that believed her and helped her report it. The courts decided since his penis was “flaccid”, it wasn’t considered an assault.
1 time	It was during her substance use disorder and she figured nobody would take her seriously.
15 years	Fear of retaliation, CPS involvement, fear for the safety of her children, and shame from family and friends
Throughout childhood	Fear of retaliation from family. Her dad, the perpetrator was a preacher
1	Was in jail at the time

### Post-intervention qualitative data

Themes emerged that easily aligned with each of the three principles of the social cognitive theory: (1) *It made me take time for myself*; (2) *It helped me to be more respectful of my body*; (3) *The connectivity …. that was helpful* (see Table 4). Each theme with supporting data is described below.

**Table 4:** Principles of SCT and exemplars

Principal of SCT	Exemplar Describing the Principle	Examples of how these factors influence self-efficacy and outcomes
Personal Factors	*It made me take time for myself*	The convenience of mHealth apps to track participant’s health was motivational to habitually use on a daily basis and assisted them in tracking their sexual health behavior more consistently than ever before.
Behavioral Factors	*It helped me to be more respectful of my body*	The positive feedback features embedded within the Nice app enhanced participant’s motivation to track trends and patterns regarding their sexual health. Further, the Nice app assisted them in identifying abusive or concerning patterns of behavior between themselves and their partners and give them confidence to talk about their concerns with their partners in a more mature manner.
Environmental Factors	*The connectivity…. that was helpful*	Participants often expressed that if they had the access and ability to use mHealth technology to manage their health and connect with a provider, it would be “helpful” and “useful” in managing their health, especially considering their roles as women and mothers. Further, they expressed anonymous chat rooms with other users could help with information exchange and support.

*Bandura, 1986; Schunk, 2020

### Theme 1: it made me take that time for myself

*It made me take time for myself*, described the first principle of the SCT, personal processes. This tenet encompasses the goals of progress which include the values central to the participants, their self-efficacy behaviors, and expected outcomes (Bandura, 1986, 1999; Schunk & DiBenedetto, 2020). Participants discussed valuing their health and wanting to be more involved with managing their health, however, barriers to healthcare and their positionality as mothers, often prevented them from seeking healthcare. This participant described her inability to access healthcare while juggling the responsibilities of being a mother while on probation:

I haven’t gone to the OB since I had my daughter eight years ago. I haven’t got a breast exam ever in my life. I’ve only attempted it on myself. I don’t go to the dentist regularly. I haven’t had a pap smear… I can’t even remember how long. I can’t even tell you if I remember how to put on a condom correctly. I’m pretty sure I know—there are things that I don’t practice, and I don’t do because, I feel like it’s not convenient…I’m in my habits. I’ve never looked at myself. Everybody else is what my concern is.

Another participant echoed this sentiment. She described how being a single mom often keeps her too busy to manage her health. However, by having access to the app, she was able to take time to track her health which was beneficial to her:

Being a single mom and having just things come up and being so busy, it made me take that time for myself and for beneficial health reasons. Sometimes you forget. These are things that you need to know because heaven forbid you do have some sort of concern regarding your sexual health or sexual activity, the app would be there to give you that information.

Collectively the sample used the Nice Sex Tracker more habitually which tracked their sexual behavior. When asked what motivated them to keep using the app, overall, convenience was a key motivating factor. All participants reported using the app primarily in the comfort of their own homes in the evening. This participant discussed how interacting with the mHealth apps became habitual, “By using it daily, it becomes something of a habit. It’s a part of your life.” Another participant shared a similar perspective, “It is convenient, especially for moms. I feel like moms mostly because sometimes we don’t have a babysitter with the kids, so it being there and available would be very convenient.”

### Theme 2: it helped me to be more respectful of my body

*It helped me to be more respectful of my body*, portrayed the second principle of the SCT, behavioral processes. This principle includes one’s choice of activity, effort, and achievement, and environmental regulation (Bandura, 1986, 1999; Schunk & DiBenedetto, 2020). The sample expressed how they enjoyed the ability to track trends and patterns regarding their sexual health. Participants often noted how the positive feedback features embedded within the Nice Sex Tracker app assisted in motivating her to track trends and her progress:

I think just seeing the progress of getting to see—I think the star ratings were really helpful for me. Getting to see like, “Oh this wasn’t as great. Why?” And so just getting to see I guess the progress of it or getting me more comfortable with even talking about those kinds of things with my partner was helpful. Just that alone was making me want to use it more.

One participant mentioned how using the mHealth apps such as the Nice app assisted with her “respecting” herself more. She stated, “I never write down my period. It’s so irregular. I don’t keep track of things. When I have these instances recorded, it just—it helps me to be more respectful of my body to remember what’s going on like, ‘Oh. I just had a yeast infection’.”

Another promising finding amongst the sample was the ability to navigate health related behavior in a more effective and confident way. This participant discussed how the use of mHealth technology allowed her to feel more “comfortable” and “mature” discussing health related issues with her partner; something that was challenging to her prior to mHealth app use. She stated:

We would be in the bedroom, we’d be talking, I would bring up the app. I would open a conversation about something that I didn’t necessarily—feel comfortable talking about with him. We got to open up a little bit. At the same time, it was weird documenting sexually specific information. It gave me a sense of having maturity, like being able to actually say that I—that this is what I did. It was empowering in a way.

In addition, several participants expressed how tracking their health daily by using the mHealth apps, they were able to see patterns in their interactions with their partners which confirmed and validated their feelings. This participant described had she had access to this technology in the past, she would have been able to identify abusive patterns which could have prevented her experiences of intimate partner abuse and violence. She described:

Any sort of anxieties that you feel afterwards. Like, ‘Hey after being with this person I experienced this sort of anxiety.’ Or ‘After being intimate with the person I had burning. I developed a UTI.’ You know and then you can as a woman be more precautious and maybe be more protective of yourself if you were sexually active and keeping track of how you felt after being with certain people. Because all of that can contribute to toxic and abusive relationships. Like, ‘This person gave me anxiety after being with them sexually.’”.

### Theme 3: the connectivity…. that was helpful

*The connectivity…. that was helpful* is the exemplar that represented the third principle of the SCT, environmental processes. This principle evaluates social factors which include instruction, feedback, standards, rewards, and opportunities for self-evaluation that assist in the achievement of self-efficacious behavior (Bandura, 1986, 1999; Schunk & DiBenedetto, 2020). Participants often expressed that if they had the access and ability to use mHealth technology to manage their health and connect with a provider, it would be “helpful” and “useful” in managing their health, especially considering their roles as women and mothers. This participant said:

It’s hard for sometimes if you have kids….to go and sit down or just to go and see a doctor in person. Whatever the case might be, it’s hard to schedule something in person. If I could just get on my phone and talk to my doctor and tell them, ‘I’m having these problems.’ I think it’d be much more helpful and useful for us women.

Moreover, participants suggested that if the mHealth apps had the ability to connect to other women, that would be effective and useful. This participant suggested in-app features to discreetly connect to other users to compare experiences. She stated a feature like that would be useful for her and possibly other women, “The connectivity. I think being able to connect to other people in the app—other people that also use the app, and maybe share certain types of information only that you want. I think that would be really, really cool.”

Another participant suggested something similar. She recommended an in-app discussion board so women can reflect and share questions and answers. She said, “Like an informational or a questions and answers [feature]. Or even an interface where you could go into a room, and you could be an avatar and you could have these discussions.” Participants stated they mostly used the Nice Sex Tracker app daily, however, had positive feedback about the uSafeUS app, which was described to be more appropriate for educational or emergency purposes. One feature the participants liked with the uSafeUS app was the ability to program into the phone a fake call or text from a trusted contact to give them the ability to get out of a troubling situation. This participant stated, “I really like the text feature on [the UsafeUS] app that way if there were a situation that I needed to get out of that I could just [say], ‘Oh my friends texting me. I gotta go.’ That was really cool.”

A few participants described the fake call or text feature would be most appropriate for them if they could speed up having their phone send a fake call or text. This participant described pre-programming well in advance a fake call or text but then enabling this feature by using a code word or phrase. She said, “If [you’re] in a situation and [if you] need to do this quick, if you could pre-program a situation and then make a code word assigned to that specific alert or that specific person or that specific scenario.”

Two participants used the uSafeUS app during acute situations. One participant described using the app to deter her from a situation that seemed as if it were about the escalate, “It was so cool because I was able to fake a call right when I needed somebody important to get me away from the situation. I can’t even tell you—my heart was fluttering. It was traumatic.” When asked what would make the app more accessible, participants voiced marketing and partnering with stakeholders to share how to download the app. One participant suggested advertising in public spaces like bathrooms, “Maybe posters posted in bathrooms with a QR code, ‘Scan this app to stay safe if you ever feel like you’re in danger on a date.’”.

Furthermore, participants were asked about their feelings regarding confidentiality. The sample gave suggestions to enhance security and protection of their personal health information. One key suggestion was to have a lock feature on the app to give an added layer of confidentiality. This participant stated, “I think a security lock on the app—that would be very helpful ‘cause my kids tend to grab my phone. Having a passcode or your face to recognize just to unlock the app [would be helpful].”

Another finding that was suggested across several participants was the implementation of an instructional video feature to assist those in navigating the mHealth apps. Although all the participants said that the apps were self-explanatory and easy to navigate once they were into the platform, they urged an instructional video may decrease women’s hesitancy when they first use the app. This participant expressed:

I would recommend a video on how to use that because there’s so many resources there, and you can overlook ‘em. I overlooked a lot of them, not because I wanted to. I just didn’t see them at the time. I feel that definitely for the uSafe app there should be a video that will tell you there’s resources for this, this, and there’s answers if you have questions. For the Nice app—I feel like the Nice app is a little bit self-explanatory.

## Discussion

The sharp and rapid increase in the number of women involved with the legal system underscores the pressing and vital need for a deeper comprehension of women’s entry points into the criminal justice system and strategies aimed at mitigating future recidivism and improving overall well-being. Women’s trajectories often originate from dysfunctional intimate relationships, experiences of abuse, trauma, victimization, and limited social and human capital (DeHart et al., 2014; Salisbury & Van Voorhis, 2009). Moreover, implementing intersectional interventions such as those that utilize mHealth technology for women on probation may become an integral component of delivering gender-responsive services (Boppre, 2019) and existing evidence suggests that focusing on these interventions may enhance outcomes (Roddy et al., 2022; Williams et al., 2021).

Evidence indicates that individuals involved with the legal system, regardless of gender, frequently have experienced multiple traumatic events, which can lead to recurring legal entanglements if left unaddressed (Givens & Cuddeback, 2021; Williams et al., 2020). Pathways to incarceration are deeply rooted in the distinctive gender-related encounters that women face in society. For instance, gender disparities concerning trauma, abuse, and victimization are extensively documented, with women influenced by the legal system reporting significantly higher rates of these experiences compared to men (DeHart & Lynch, 2021). All ten participants had disclosed some form of violence or abuse in the form of physical, sexual, verbal, psychological, or financial leading to their initial arrest. Participants stated if had they had access to a wearable intervention such as an mHealth app to track their feelings, symptoms, and outcomes, they would have been better prepared to identify such abusive patterns and warning signs and would have bene better equipped to report it.

Further, data is suggestive that women are more willing to engage in technology to manage their health even if they are novice at mHealth technology (Crawford et al., 2023a; Heron et al., 2019). We found women, particularly mothers, are adaptable to using technology to manage their health if it allows them to balance their other roles as caregivers more effectively. The participants in our study reported using mHealth technology, despite this intervention being new to them, was usable and feasible because of its convenience which allowed them to juggle the demands of their probation and roles as caregivers to young children.

Furthermore, in our initial study, women on probation voiced their desire to access health and safety-related resources to help enhance their wellbeing, however, these women also described they felt stigmatized or were fearful of rearrest (Crawford et al., 2022a, b, 2023b). Therefore, women’s health and safety disparities often went under reported and/or under treated while on probation (Crawford et al., 2022a, 2023b). Participants in this study expressed similar concerns. They verbalized that having a way to learn about their health and track their behavioral patterns in a discreet way empowered them to make better choices and to have more respect for their bodies and overall wellbeing.

Yet, participants in preliminary studies have described how interpersonal relationships with their children or significant others motivated them to maintain healthy behaviors (Crawford et al., 2022a, 2023b). Studies that are related to reentry, treatment and rehabilitation have suggested the same motivating factors for women in the community; the more they are connected and engaged with their families and children, the more motivated they are to commit to recovery (Adams et al., 2021; Hoff et al., 2021; Crawford et al., 2022b). The same was true with the findings of this study. Women found value in the app’s abilities to keep them abreast of their health so they can be more present with their children and have a better quality of life.

Women have expressed wanting resources that embrace their role as caregivers and that also assist in the management of their health conditions (Crawford et al., 2022b). Further, women in preliminary studies have described being part of a community and support network has assisted in strengthening their coping strategies (Thomas et al., 2019); Crawford et al., 2023b). These data validate the findings of this exploratory intervention development study. Participants enjoyed the ability to have a tool to make managing their health more effective and convenient that was on their own terms and managed by themselves in the safety of their homes. Finally, social determination theory is a theory that describes how individuals will have better self-management of their health, ownership over their progress, and commitment to changing their behavior if they have autonomy over their bodies and plan of care (Thomas et al., 2019). Our findings were reflective of this with women engaging in their health management more and stating they had more “respect” over their bodies after using mHealth technology to manage their health on their own and on their own terms. Therefore, we anticipate an autonomous-supportive communication strategy using mHealth technology for women on probation to manage their health and safety may enhance self-efficacy by allowing them to have more autonomy over their progress and treatment choices.

### Limitations

Although the findings of this study are promising, we must acknowledge the inherent limitations. We purposely sampled women on probation within South-Central Texas, a predominantly Hispanic region that limits generalizability. Yet, purposely sampling from this region gave us substantial insight on a demographic of women (Hispanic) who are largely limited in current research. Further, the methods relied on self-report and recall of the participants to input their health information into the mHealth apps. This limitation was unavoidable, however, the discreet nature and readily available intervention such as an mHealth app on participant’s mobile devices may have alleviated possible bias.

### Major implications

Intervention development in the form of mHealth applications for women on probation to access using their mobile devices is a feasible and necessary intervention. There are tremendous barriers to safe, affordable, non-judgment, and accessible health care for women who are in the community with legal system oversight (Crawford et al., 2022b). Thus, streamlining services in a discreet and time-sensitive way can be pivotal in breaking barriers for populations that need it most. We suspect the use of mHealth technology will empower women on probation to have shared decision making and autonomy over their healthcare to promote commitment and self-efficacy in managing their health. Partnerships are needed on all levels within and outside the criminal justice system, academia, policy, and those with lived experience must work together to implement such community-based interventions and resources for women on probation. Lastly, steps much be taken to use a community participatory research and reproductive justice approach by centering women’s experiences and needs by having interventions informed by those with the lived experience – women on probation.

## Conclusion

MHealth apps give the ability to track symptoms and patterns of behavior specific to women’s health and safety in a discreet, convenient, and effective manner that may reduce morbidity, mortality, and recidivism in women on probation. This research suggests that a culturally tailored mHealth app may be an appropriate intervention to provide timely gender-responsive feedback, resources, and health care to women on probation. The long-term goal is to improve health and safety self-management behavior and outcomes, prevent violence, and reduce recidivism in women who are on community supervision.

## Data Availability

Raw data were generated at the University of Texas Health Science Center at San Antonio. Derived data supporting the findings of this study are available from the corresponding author A.D.C. on request.
